# Concepturealize™: a new contribution to generate real-needs-focussed, user-centred, lean business models

**DOI:** 10.1186/s13731-022-00198-4

**Published:** 2022-01-25

**Authors:** Graeme Joseph Allen

**Affiliations:** Centre for Innovation, Technology and Entrepreneurship, Institute for Systems and Computer Engineering, Technology and Science (INESC TEC), R. Dr. Roberto Frias, Porto, Portugal

**Keywords:** Business model design, Customer development, Design Thinking, Entrepreneurship, Innovation, Innovation management, Lean Startup, Value proposition

## Abstract

It is widely accepted that somewhere in the region of 90–95% of startups fail. It is often suggested that the majority of unsuccessful startups either failed to identify a viable idea, or they failed to execute the idea effectively enough to get to market before running out of cash. Two approaches stand out as being particularly well-suited to addressing these problems: Design Thinking and Lean Startup, respectively. This paper presents the Concepturealize™ methodology that cross-applies Design Thinking and Lean Startup as a single iterative process and that enables the entrepreneur to generate real-needs-focussed, user-centred, lean business models. Existing literature reveals a need for further exploration of cross-application of Design Thinking and Lean Startup (and other related methodologies) in the areas of business model development and innovation. This work answers the research question by review of prior attempts to combine Design Thinking and Lean Startup and presenting the Concepturealize™ model that cross-applies Design Thinking and Lean Startup in a single iterative methodology and that enables the practitioner to generate real-needs-focussed, user-centred, lean business models. By following this new process model correctly, a practitioner will be guided to uncover a viable way to create value, develop a deep understanding of the value proposition, the target customers and how to reach and serve them, together with the expected revenue and costs, all needed to properly formulate the business model. Finally, the practitioner may use the Concepturealize™ model to retest the problem–solution fit and understand how the customers perception of value has altered, each time a new product or new features are launched, looking to continually add value at each cycle. Whilst prior research has explored how organisations may make use of both DT and LS, it has failed to demonstrate how they may be used in parallel, throughout the entire business model development process, instead it demonstrates examples of insight into where to transition from one model to the other. This work progresses the state of the art by following Design Science guidelines to present how the true, in-parallel, cross-application of DT and LS, in the context of business model development, is possible.

## Introduction

Innovation is essential for achieving and maintaining a sustainable competitive advantage, both for startups and established businesses, alike (Crossan & Apaydin, 2010; Foss & Saebi, 2018; Prajogo, 2016).

According to CB Insights (2019), a lack of market need is the leading reason for failure of startups, noted in 42% of cases from a post-mortem of 101 failed startups. Running out of cash is the second most cited reason at 29%. Cantamessa et al., (2018) conducted an analysis of a database of 214 startup failure reports. They identified the most common reasons for failure are a missing or incorrect business model (35%), lack of business development (28%), running out of cash (21%), and no product–market fit (18%). This suggests that the majority of failed startups either failed to identify a viable idea, or they failed to develop a business model to enable execution of the idea effectively enough to get to market before running out of cash. Two approaches stand out as being particularly well-suited to addressing these problems: Design Thinking and Lean Startup, respectively.

### Design Thinking (DT)

The basic concept of DT is to take a designer’s approach to creativity and innovation in business (Brown, 2008; Liedtka, 2011). DT is an approach that takes real user-needs and matches them with solutions that are technically feasible and are viable for creating value and market opportunity (Lewrick et al., 2018; Liedtka, 2014). DT was adapted for business purposes by David Kelley, founder of IDEO^1^ (Kelley & Kelley, 2015). The popularity of the DT approach was helped by its adoption by the d.school at Stanford University (Lichtenthaler, 2020) and by further development by the Hasso Institute (Hasso et al., 2009; Lichtenthaler, 2020).

### Lean Startup (LS)

LS can be defined as a blueprint for how to run a startup. Essentially, the goal is to find a product–market fit by moving a minimum viable product (MVP) through the build–measure–learn feedback loop (Ries, 2011). The LS model incorporates Customer Development^2^ and Lean Manufacturing^3^ and makes use of Business Model Design (BMD) as well as tools such as Innovation Accounting, Split Testing, Five Whys and Business Model Canvas (Blank, 2012; Osterwalder & Pigneur, 2010; Ries, 2011).

Despite increasing popularity of the practise of DT and LS, independently, prior research exploring the advantages and implications of using both approaches together is limited (Koen, 2015; Lichtenthaler, 2020; Müller & Thoring, 2012). When organisations rely on DT without LS, there is a relatively high likelihood of developing a promising idea (Lewrick et al., 2018), however, it is likely that there will be challenges, or at least, limited efficiency in commercialisation and execution when the innovation process is based on traditional approaches such as Stage-Gate®.^4^ Conversely, if organisations rely on LS without considering DT, there is a relatively high likelihood of achieving success in developing a minimum viable product (MVP) and in reducing time to market (Ries, 2011), however they may lack in the consideration of the superiority of other ideas. This is due to the fact that LS usually assumes that the initial idea is contained within the founders’ vision (Koen, 2015; Müller & Thoring, 2012). If, on the other hand, organisations use DT and LS together, there is a relatively high likelihood of achieving promising ideas to solve real customer-needs, with relatively short time to market and high level of flexibility that comes from the iterative foundations of both models (Lichtenthaler, 2020).

Although different models, there is some overlap in the processes of DT and LS, therefore, it may not be the most efficient approach to use both models in full. A symbiotic relationship between DT and LS could capitalise on the broader capacity of DT and take a holistic approach towards innovation, not just to develop a product prototype or MVP, but also to drive innovation across all aspects of the startup’s strategy; iteratively feeding the outputs of these innovation efforts into each element of the LS method, creating a more robust, better-tested, and user-centred business model with a value proposition that addresses real (implicit) customer needs.

### Literature gap

A review of the published literature reveals increasing popularity of using DT and LS, either independently, or sequentially in near-isolation as separate methods, with focus on using DT for product or service design and on using LS for building the business model to exploit the product or service. Several attempts have been made to combine the methodologies, demonstrating problem relevance. Of the studies within the literature review that have presented a new process model, none have proposed a fully integrated cross-application of LS and DT—instead, they define a point to transition from one model to the other.

The literature reveals an interest in combining elements of DT with those of LS (as well as with elements from other process models) to promote needs-focussed, user-centred innovation. The literature also reveals a need for further exploration of true cross-application of DT and LS in the context of business model development.

### General research objective

To answer the research question, how can DT principles be combined into LS to generate real-needs-focussed, user-centred, lean business models?

### Specific research objectives

1. To demonstrate how the true, in-parallel, cross-application of DT and LS, in the context of BMD, is possible.
2. To present a novel methodology for BMD that improves on the independent use of both DT and LS, whilst retaining the lean nature of LS and the user-centredness of DT.

## Background

### Design Thinking vs. Lean Startup

DT is an approach that starts with real user-needs and takes a designer’s approach to find solutions that are technically feasible and viable (Lewrick et al., 2018; Liedtka, 2014), whereas the LS model is built upon Customer Development, which at its very foundation, makes the assumption that most startups are technology-driven—they are founded and funded by visionaries that already have a product or service idea and now need to find customers and markets (Blank, 2005, 2012).

LS incorporates Lean Manufacturing—a methodology developed by Taiichi Ohno and Shigeo Shingo at Toyota, that gave rise to the ‘lean revolution’ and that lends its name to LS (Ries, 2011). The principles of lean are to identify value, map the value stream, create flow, establish pull, and create perfection (Womack & Jones, 2003). In practice, it makes use of techniques such as drawing on the knowledge and creativity of individuals, the shrinking of batch sizes, just-in-time inventory control and production and a reduction of cycle times (Womack et al., 1990). At its heart, the goal of being lean is simply to eliminate waste. LS adapts these ideas to the context of entrepreneurship, proposing that entrepreneurs measure their progress differently from the way other organisations do. As progress in lean manufacturing is measured by the production of high-quality physical products, LS uses validated learning (Ries, 2011). BMD defines a business model as the blueprint of how a company does business by serving as a plan that allows the design and realisation of the business structure and systems that form the company’s operations and structure. “It is the translation of strategic issues, such as strategic positioning and strategic goals into a conceptual model that explicitly states how the business functions.” (Osterwalder et al., 2005, p. 4).

**Fig. 1:**
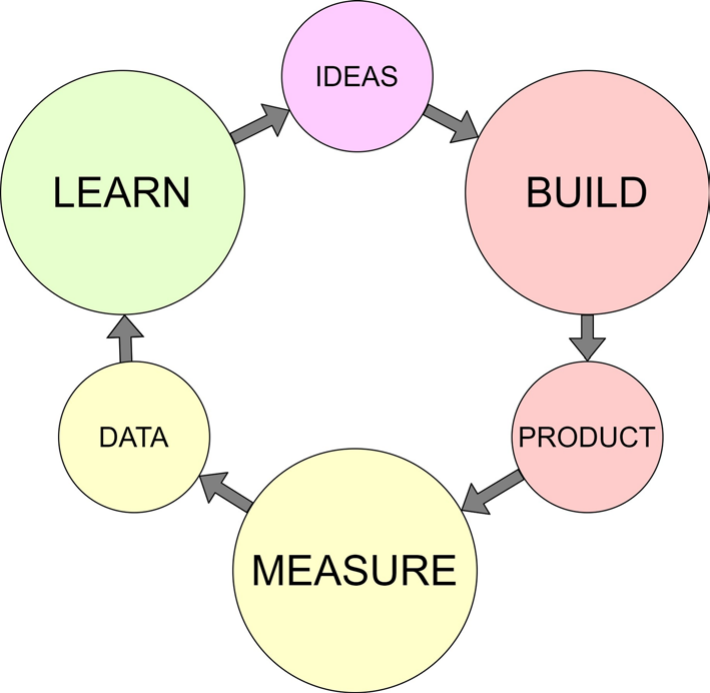
Build–measure–learn feedback loop (adapted from Ries, 2011, p. 75)

At the core of the LS model is the BML feedback loop. The BML feedback loop is a lean approach to finding the validated learning required to ensure the startup offers value and achieves growth. An important note is that whilst performed as build–measure–learn, the cycle should be planned in reverse, that is to understand what needs to be learned, then what data to measure to ensure validated learning, and finally the form of the MVP required to run the experiment. The practitioner would begin by identifying the hypotheses to test, then the metrics to test them against, and then plan the minimum set of features required for the MVP to enable the data to be sourced. This MVP should be the version of the product that allows for a full turn of the BML feedback loop with the least amount of effort and least amount of time (Ries, 2011). An abstract diagram is shown in Fig. 1.

According to Tim Brown, CEO of IDEO, “Design Thinking is a human-centred approach to innovation that draws from the designer's toolkit to integrate the needs of people, the possibilities of technology, and the requirements for business success” (Brown, 2008).

Historically, design was considered a downstream process to create a polished wrapper to put around an idea to help market it to customers. Now, however, rather than asking designers to make an already developed idea more attractive to consumers, companies are asking them to create ideas that better meet users’ needs. The former role is tactical, and results in limited value creation; the latter is strategic and leads to dramatic new forms of value (Brown, 2008). Additionally, as economies shift from industrial manufacturing to knowledge and service delivery, innovation’s scope is expanding. Its objectives are no longer just physical products; they are new sorts of processes, services, interactions, ways of communicating and collaborating—exactly the kinds of human-centred activities in which DT can make a decisive difference (Brown, 2008).

**Fig. 2:**
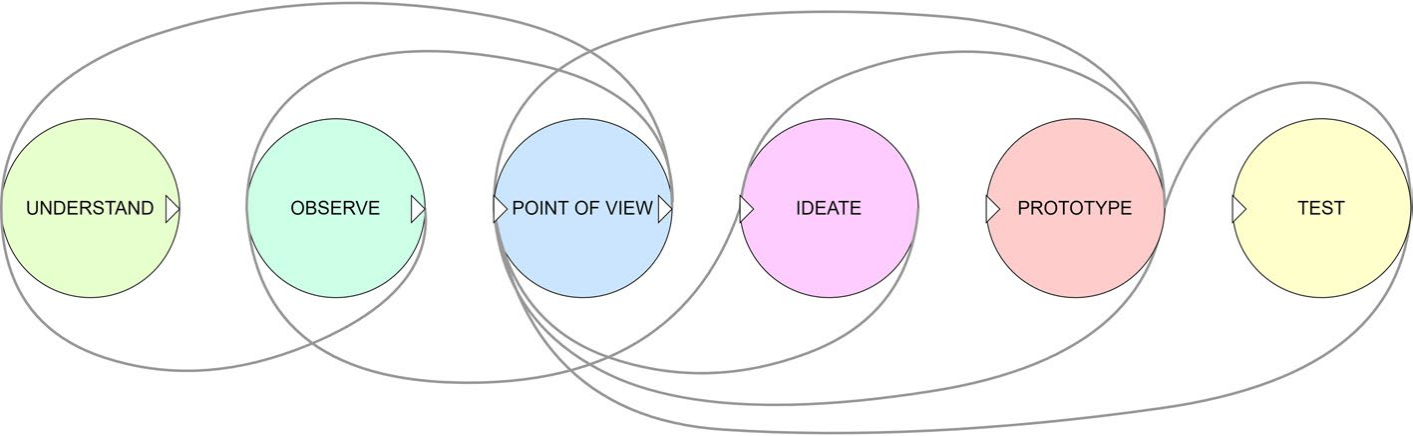
DT abstract process model ( adapted from Hasso et al., 2009, p. 220)

Key aspects of DT include a focus on a dynamic approach to problem solving—working particularly well on poorly bounded problems by utilising prototyping and iteration for rapid learning; an approach towards problem finding—finding leverage in re-framing problems and using ethnographic and empathic research to define the ‘problem space’, avoiding symptoms, and identifying implicit needs over explicit needs; and the use of a human-centred co-creation process, focussed on real end-user needs (Kelley & Kelley, 2015). An abstract process model, representing the DT process is shown in Fig. 2.

### Lean Startup and Design Thinking similarities

**Fig. 3:**
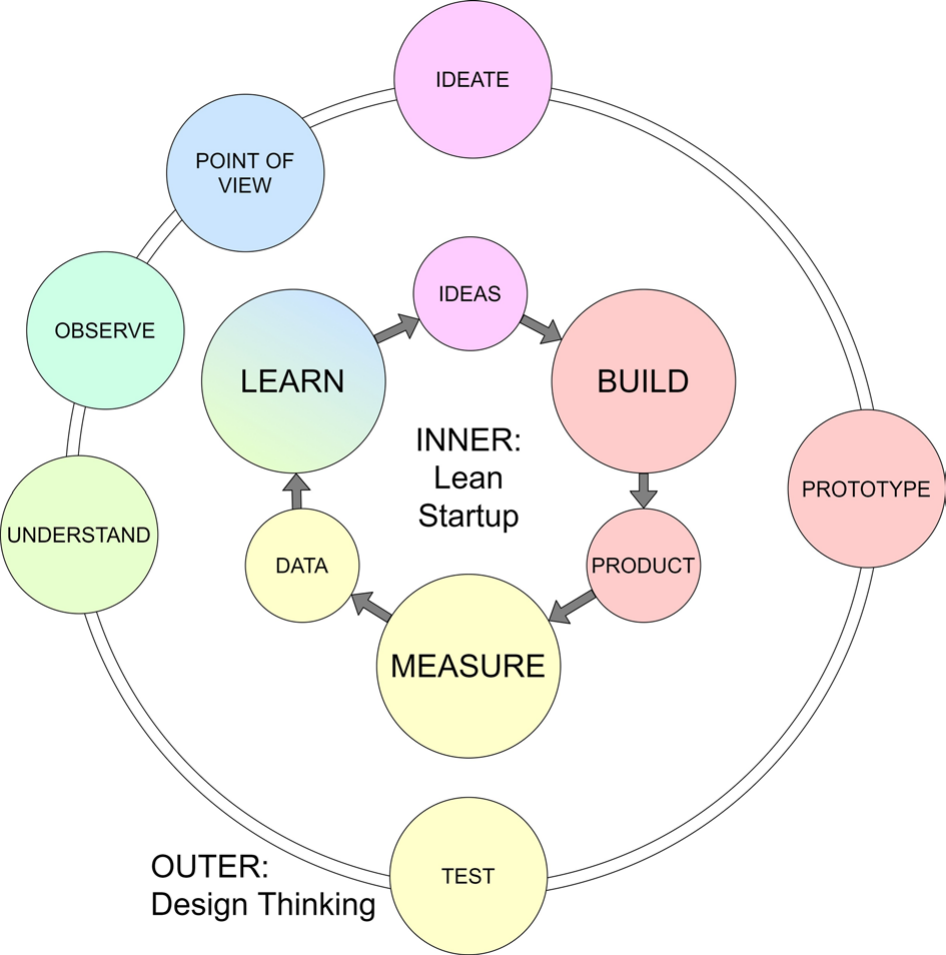
Comparison between LS BML and DT process model (adapted from Hasso et al., 2009, p. 220; Ries, 2011, p. 75)

Similarities between LS and DT can be seen by comparing the abstract process models shown in Figs. 1 and 2. ‘Ideas’ in LS can be considered to equate to ‘Ideate’ in DT; similarly, ‘Build’ and ‘Product’ in LS to ‘Prototype’ in DT; ‘Measure’ and ‘Data’ in LS to ‘Test’ in DT; and ‘Learn’ in LS to ‘Understand’, ‘Observe’ and ‘Point of View’ in DT. This is illustrated in Fig. 3.

Aside from the similarities between the LS build–measure–learn and the DT process model, as described above, there are several other key aspects and assumptions that LS and DT have in common, namely:

**Goal.** Both DT and LS have innovation as the main goal.

**Approach.** DT refers to a ‘user-centred’ approach whereas LS takes a customer-oriented (customer-centric) approach. Although subtly different, these approaches are similar in nature.

**Uncertainty.** DT assumes ‘wicked problems’, i.e. a problem that is unclear, complex in nature, non-linear in cause, and difficult to solve (Rittel, 1972), and LS assumes an unclear customer problem.

**Testing.** DT refers to ‘failing early’ and LS refers to ‘failing fast’. Both on the premise that the sooner it is realised that an idea is identified as not working, the sooner it can be updated and retested.

**Iteration.** DT has iteration at its core, as does LS with the BML feedback loop (pivoting).

**Prototype testing.** ‘Prototype’ in DT and minimum viable product (MVP) in LS.

**Rapid iteration.** In both models, prototype/MVP testing and iteration with a ‘fail fast’ credo result in rapid iteration.

**Target group.** Both models focus on users and other stakeholders. LS distinguishes between the different stakeholder types (customers, users, recommenders, influencers, economic buyers and decision-makers), whereas DT does not refer directly to market typology (Müller & Thoring, 2012).

### Lean Startup and Design Thinking differences

The differences, and in some cases clashes, between DT and LS demonstrate that rather than necessarily opposing each other, the models could be used to complement each other by filling the gaps. The major differences between DT and LS are shown in Table 1.

**Table 1:** Major differences between DT and LS

Aspect	Design Thinking	Lean Startup
Scope and focus	Scope extends to general innovation, without bounds to the purpose or subject	Strongly focussed on high-tech product or service innovation and BM innovation within startup companies
Ideation	Has ideation as a key part of the iterative process, the project starting with a challenge, not the idea	Assumes the idea comes with the founders’ vision
Qualitative methods	Strong focus on qualitative methods with ethnographic research, observations, empathic research, etc.	Focusses less on qualitative research in favour of quantitative methods
Quantitative methods	Focusses less on qualitative research in favour of qualitative methods	Strong focus on quantitative methods including metric-based analysis, matrices, innovation accounting and metrics for the ‘engines of growth’ (viral, sticky and paid) (Ries, 2011), etc.
Business model	Does not focus on BM creation and would only assist with BM innovation if specifically utilised as such	BM creation and BM innovation are strong focus points of LS
Adaption of deployments	Does not focus on adaption of deployments	Looks back to its roots at Toyota and draws form the famous ‘Andon Chord’, which allowed any worker to ask for help as soon as they identified a problem; stopping the entire production line, if necessary (Ries, 2011). Five Whys method is used to identify the cause of failure and enable rapid rectification
Hypothesis testing	Practitioners may use hypotheses as part of the process; however, the cycle begins with a challenge, or ‘wicked problem’ rather than the hypothesis itself	The Build element of the BML Feedback Loop is based upon a hypothesis, therefore the Measure and Learn steps are the testing of this hypothesis

### Summary

Although there are several distinct differences between the models, including scope, methods, and outputs, both models have similar goals and target groups and both achieve those goals through rapid, iterative testing and measuring/learning, Ultimately the aim of both LS and DT is to innovate solutions to problems through an iterative approach, centred on the user and/or customer. Due to the overlap in the processes of DT and LS, it may not be the most efficient approach to use both models in full. Creating a symbiotic relationship between DT and LS could capitalise on the benefits of both models, however neither model presents an explicit method for doing so.

## Research method

The work discovers the research question through a thorough literature review, the question is answered by developing a new business model creation methodology (named Concepturealize™) to cross-apply DT and LS with each other.

The methodology selected to achieve this was based on the seven guidelines of design-science research, namely “1. design as an artefact; 2. problem relevance; 3. design evaluation; 4. research contributions; 5. research rigour; 6. design as a search process; and 7. communication of research” (Hevner & Park, 2004, p. 83). The approach taken to fulfil the design-science guidelines is shown in Table 2.

**Table 2:** Approach taken towards the design-science guidelines

Guideline	Approach and evidence
1.Design as an artefact	The research presents a viable artefact in the form of the Concepturealize™ methodology
2.Problem relevance	Problem relevance is demonstrated by the amount of interest in cross-applying DT and LS discovered in the knowledge base Input from target users informs the design of the artefact as well as validating problem relevance Observation of aspiring entrepreneurs in an entrepreneurial educational setting Seeks critical feedback from target users
3.Design evaluation	Analytical (static analysis): examines artefact structure and elements for static qualities (comprehensiveness and applicability to the problem, integrity of the toolset, familiarity of individual tools to target users, and ease of use) Descriptive (informed argument): artefact builds upon existing artefacts with demonstrated utility Descriptive (scenarios): artefact utility demonstrated through detailed scenario
4.Research contributions	High importance given to novelty (applying existing knowledge in a new way), generality (applicable to entrepreneurs in all sectors) and significance (provides significant improvements over the singular use of existing methodologies)
5.Research rigour	Comprehensive and structured review of the knowledge base
6.Design as a search process	Iterative approach to designing the artefact with static analysis and target-user input feeding iteration cycle
7.Communication of research	Publication of research and artefact

### Literature review

To discover the research question and to ensure proper rigour, an in-depth literature review following the Preferred Reporting Items for Systematic Reviews and Meta-Analyses (PRISMA) method was conducted (Moher et al., 2009) (in design-science, rigour is derived from effective use of the knowledge base (Hevner & Park, 2004)). The aim of literature review was to discover prior work with a focus on combining or cross-applying DT and/or LS, either with each other or with any other process model or methodology, and identified several studies in which some form of hybrid process model or methodology was created that combines LS and DT, either with each other or with another model. The review was conducted on all document types, from all years, contained within the SCOPUS or Web of Science databases. The following is a summary of the keywords and search criteria used to discover articles relevant to the research topic on the two databases used (Web of Science and SCOPUS).

#### Web of science

Topic (Title, Abstract, Author Keywords, Keywords Plus): (“Design Thinking” OR “Lean Startup")); timespan: all years; indexes: SCI-EXPANDED, SSCI, A&HCI, CPCI-S, CPCI-SSH, ESCI, CCR-EXPANDED, IC; results: 2,215.

#### SCOPUS

Article title, Abstract, Keywords: “Design Thinking” OR “Lean Startup"; all years; all document types; all Access types; results: 3,629.

### Literature review meta-analysis

The search of both databases yielded 5844 results, of which 1,659 were duplicates. The 4,185 unique items were screened by title and abstract and 4,017 were excluded for not containing reference to at least two methodologies, ideologies, or process models. The full content of the remaining 168 articles was read and a further 88 articles were excluded for neither: (a) discussing the combination or cross-application of one process model or methodology with any other process model or methodology; nor (b) having a strong focus on DT or LS. Finally, the remaining 80 articles were reviewed in greater depth and 36 were excluded for not having either the combination or cross-application of models, nor DT or LS, as their primary focus.

### Novel hybrid models and methodologies in literature

From the remaining 44 articles, seven studies focus on the cross-application of LS and DT, either with each other or with another model; and of these, five present a novel process model or methodology. These studies are presented in Table 3.

**Table 3:** Novel hybrid models and methodologies in literature

Paper title (author, year)	Approaches/models covered	Summary	Testing/validation
Design Thinking vs. Lean Startup: a comparison of two user-driven innovation strategies (Müller & Thoring, 2012)	Design Thinking, Lean Startup	Based on 1) published literature and case studies, and 2) process models for the two different processes Improve DT by implementing feedback testing and iteration (LS pivot) earlier in the process, before prototype); implement quantitative methods from LS; develop BM in addition to prototype Improve LS by introducing qualitative methods from DT (e.g. ethnographic); adopt DT synthesis methods; adopt DT use of personas "Lean Design Thinking": DT understand, observe, point of view, ideation + DT prototyping merged with LS customer discovery + LS customer validation + testing after each step (incl. both qualitative and quantitative methods)	Conceptual only. Not tested
Bridging sustainable business model innovation and user-driven innovation a process for sustainable value proposition design (Baldassarre et al., 2017)	Sustainable business model innovation, User-driven innovation (incl. DT and LS)	"Sustainable Value Proposition Design": Iterative process with roots in LS and DT (Talking, Thinking, Testing) to design environmentally sustainable value propositions	Implemented in a design project to develop a value proposition to trigger energy saving behaviour in commercial office buildings
The best of three worlds—the creation of Innodev a software development approach that integrates Design Thinking, Scrum and Lean Startup (Dobrigkeit & De Paula, 2017)	Design Thinking, Scrum, Lean Startup	"InnoDev": a three-phase software development process combining elements from Design Thinking, Scrum and Lean Start-Up	Conceptual model only. Not tested
A process model integrated to innovation management tools to support technology entrepreneurship (Souza et al., 2018)	Lean Startup, Scrum	"P-Start": "a seven-step process model integrated to innovation management tools to support entrepreneurs in the context of startup creation and development"	Tested over 27 months with three startups
Software project management combining agile, Lean Startup And Design Thinking (Ximenes et al., 2015)	Agile, Lean Startup, Design Thinking	"Converge": Agile software development of MVP developed in LS BM. Challenges, referred to as "knots", addressed using DT techniques	8-week project within team of undergraduate students
Skip the silver bullet: driving innovation through small bets and diverse practices (Grossman-Kahn & Rosensweig, 2012)	Design Thinking, Lean Startup, Agile	“Discovery by Design™” model for innovation—a system developed by the Nordstrom Innovation Lab to integrate multiple approaches of innovation: DT to provide a roadmap to creative and human-centred solutions. LS to focus on building the right thing for the customers and to give a framework for delivering validated learning. Agile & Lean to optimise the process and enable to move quickly	Tested by Nordstrom Innovation Lab to develop and experiment with ideas within the Nordstrom retail business
Agile innovation the complementarity of Design Thinking And Lean Startup (Lichtenthaler, 2020)	Agile, Design Thinking, Lean Startup	Discussion of use of Agile, DT and LS but no attempt to combine into single model	No novel model presented. Examination of complementarity between approaches only
The coexistence of Design Thinking and stage-and-gate in the same organisational context: challenges and need for integration (Franchini et al., 2017)	Design Thinking, stage-and-gate	A single case study of a food company where DT and stage-and-gate methods co-exist	No novel model presented. Case study of coexistence

#### Lean Design Thinking

In “Design Thinking vs. Lean Startup: A Comparison Of Two User-driven Innovation Strategies”, Müller and Thoring (2012) describe a process model that combines elements from DT with elements from LS. The model, known as “Lean Design Thinking”, incorporates understand, observe, point of view, ideation from DT; prototyping (DT) merged with customer discovery (LS); customer validation (LS); and includes testing after each step (including both qualitative and quantitative methods of testing). As such, rather than integrating DT into LS, Lean Design Thinking borrows elements exclusively from DT for the ‘understand’, ‘observe’, ‘point of view’ and ‘ideation’ stages. The process then hands the output over LS during ‘prototyping/customer discovery’ stage, from whereon all elements are borrowed exclusively from LS (with the exception of the suggested use of both qualitative testing and metric testing at each stage). In summary, Lean Design Thinking does not combine DT with LS, rather it suggests a point to transition from DT to LS, as well as suggesting to apply both qualitative testing and metric testing at each stage of the process(es).

#### Sustainable value proposition design

Baldassarre et al. (2017) describe a new iterative process model intended to enable the design of environmentally sustainable value propositions. It combines Sustainable Business Model Innovation with User-driven Innovation (described by Baldassarre et al. (2017) as including LS and DT). The model combines the iterative processes from LS and DT. Sustainable Value Proposition Design was tested in a design project to develop a value proposition to trigger energy saving behaviour in commercial office buildings and has a rather tight focus on integrating environmental sustainability objectives into business models.

#### InnoDev

Dobrigkeit and De Paula (2017) integrate elements from DT, Scrum and LS to create a new process model for software development. InnoDev is described as a three-phase model, consisting of (1) a DT phase, (2) an initial development phase and (3) a development Phase 6. Phase 1 of InnoDev follows the DT process to explore the problem and solution and define a product vision. Phase 2 redefines and develops the product vision into a proof-of-concept prototype, following the idea of an MVP from LS; metrics such as the AARRR funnel are used in this phase. In phase 3, the MVP is tested and extended (and pivoted when necessary), following the concepts of the LS BML feedback loop, with the team making use of the concepts of Sprints and Backlog concepts from Scrum. DT breakouts occur on an ad hoc basis in response to problems or blockers related to the product.

#### P-Start

"A seven-step process model integrated to innovation management tools to support entrepreneurs in the context of startup creation and development" (Souza et al., 2018), P-Start combines elements of LS and Scrum. The seven steps of P-Start are (1) planning and organisation; (2) problem identification and testing; (3) concept development and testing; (4) sales preparation and testing; (5) product testing and maturation; (6) commercial expansion; and (7) consolidation and renewal. It should be noted that P-Start is not designed as a linear process, but a cyclical one; each step being intended to be used as a tool to be applied as appropriate to guide decision-making and prioritisation. P-Start makes use of Scrum methods to “strengthen tactical management of startup processes, marked by high uncertainty levels, complex problem solutions and cooperation” (Souza et al., 2018), with the product backlog tool being central to the process model.

#### Converge

Developed by Ximenes et al. (2015), Converge takes elements from Agile, LS and DT. Converge was designed “to be applicable to development teams in need of creative solutions” (Ximenes et al., 2015, p. 357). The Converge model employs the Lean Canvas and other tools used in LS, such as the 5-whys and integrates them with the DT flow, as well as Agile concepts and Extreme Programming elements such as pair programming and collective code ownership.

Table 4 represents a comparison between the main features of DT, LS and the five models described above (Lean Design Thinking, Sustainable Value Proposition Design, InnoDev, P-Start and Converge). It can be seen that each of the features that are used by both DT and LS (i.e. assumes uncertainty; prototype/MVP; iteration/pivot focus; rapid iteration; and user-centred) are shared by all five of the other models. In addition, all five models make use of quantitative methods for testing.

**Table 4:**
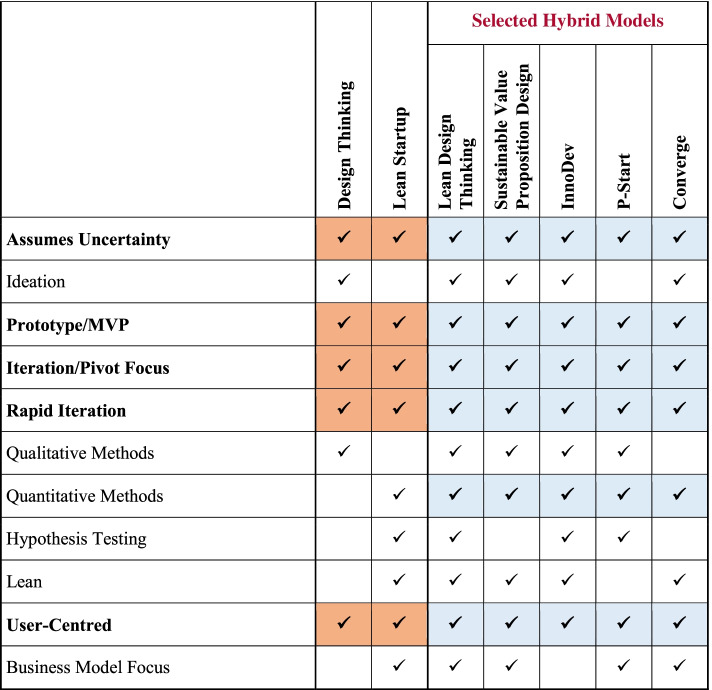
Comparison of features; DT, LS, and other identified models

Orange colour signifies features common to both DT and LS, blue colour signifies features common to all hybrid models

Other work has studied combining DT or LS with other methodologies, for example, Franchini et al. (2017) explored a single case study where DT was combined with stage-and-gate for new product development within an established food and beverage company. Bicen and Johnson (2015) recommend a further study to explore the qualities of lean innovation capability and the nature of its ties with DT methodology; Laursen and Hasse (2019) propose a need to identify and unfold methodological approaches for DT; and Baldassarre et al. (2017) identify a need to assess the application of business model co-creation in the different stages of the innovation process. Lichtenthaler (2020) discusses the benefits of co-adoption of DT and LS practices and refers to some examples of success in doing so, for example by the sports equipment manufacturer, Adidas with its ‘Speedfactory’ initiative. However, the paper does not attempt to create a new process model for such. Grossman-Kahn and Rosensweig (2012) discuss Discovery by Design™, which integrates multiple approaches of innovation: DT provide to provide a roadmap to creative and human-centred solutions; to uncover latent needs, and generate innovative solutions that are desirable, feasible and viable. Use of LS to focus on building the right thing for the customers, providing a framework for delivering validated learning with tools like BML and innovation accounting. Agile and lean to optimise the process and to enable to move quickly. DT enables the practitioner to know what to build—agile is how they build. Like Lean Design Thinking, Discovery by Design™ does not combine DT with LS, but it suggests a point to transition from DT to LS (and Agile). The paper does not go into detail about how the Discovery by Design™ model for innovation should be prescribed, however it demonstrates that such an approach may support the need for responsive innovation even within large organisations.

### Reflection

Review of the published literature reveals increasing popularity of using DT and LS, either independently, or sequentially in near-isolation as separate methods, with focus on using DT for product or service design and on using LS for building the business model to exploit the product or service. Several attempts have been made to combine the methodologies, as discussed above, demonstrating problem relevance, for example, Müller and Thoring (2012) propose a hybrid process model that they refer to as ‘Lean Design Thinking’, although it does not combine DT with LS, rather it suggests a point to transition from DT to LS. Lichtenthaler (2020) discusses the benefits of co-adoption of DT and LS practices but does not attempt to create a new process model for such. Of the seven studies, within the literature review, that have attempted to develop a new process model, three recommend further research by applying the model to further testing (Baldassarre et al., 2017; Dobrigkeit & De Paula, 2017; Müller & Thoring, 2012), and two studies recommend studying the application of the model to different settings, for example organisational structure or business maturity (Souza et al., 2018; Ximenes et al., 2015).

Each of the five features that are used by both DT and LS (i.e. assumes uncertainty; prototype/MVP; iteration/pivot focus; rapid iteration; and user-centred) are shared by all five of the models previously described. In addition, all five models included in Table 4 make use of quantitative methods for testing.

The literature reveals an interest in combining elements of DT with those of LS (as well as with elements from other process models) to promote needs-focussed, user-centred innovation. The literature also reveals a need for further exploration of true cross-application of DT and LS in the context of business model development.

### Research question

Previous attempts to cross-apply DT with LS either fail to fully combine the DT principles with LS (instead, suggesting point to hand the DT-born idea over to LS for execution) (Müller & Thoring, 2012), have a narrow focus (i.e. Baldassarre et al. (2017) focus on environmental sustainability, and Dobrigkeit and De Paula (2017) and Ximenes et al. (2015) focus on software development), or they do not retain the lean nature of LS (Souza et al., 2018)—raising the big question, how can DT principles be combined into LS to generate real-needs-focussed, user-centred, lean business models?

## Creating a new methodology

Building on LS, DT and the work of Müller and Thoring (2012), Baldassarre et al. (2017), Dobrigkeit and De Paula (2017), Souza et al. (2018) and Ximenes et al. (2015), the Concepturealize™ methodology was devised. The design process of the Concepturealize™ methodology follows the design-science approach (Hevner & Park, 2004).

The previous attempts to generate new hybrid process models or methodologies were classified according to the level of testing rigour (i.e. whether used in real-world case studies); the level of success of the model; and where available, evidence of adoption of the model, post-study. The most developed and tested models were then used as informed argument to build a foundation for Concepturealize™ as a working artefact (design-science guideline 1: design as an artefact), designed to address the research question (design-science guideline 2: problem relevance). For example, each of the six features that are shared by all five hybrid models (assumes uncertainty; prototype/MVP; iteration/pivot focus; rapid iteration; use of quantitative methods; and user-centredness) were built into the first iteration of Concepturealize™; “Lean Design Thinking” seeks overlap between DT and LS, so this was explored in the creation of Concepturealize™ in order to enhance the leanness of the methodology; and “Converge” integrates LS elements directly into the DT flow, so this was attempted in early iterations of Concepturealize™ before moving to a bi-directional integration, following user feedback.

The lessons learned from the previous attempts, as well as the literature pertaining to DT and LS, and other relevant models, were used to inform the creation of the Concepturealize™ methodology. An important note is that a design-science approach to organisational projects, such as this one, must be specific in terms of defining the desired nature and level of improvement (Van Aken, 2007). Therefore, the scope and depth of this literature review has a direct input on the success of the project (design-science guideline 5: research rigour).

**Fig. 4:**
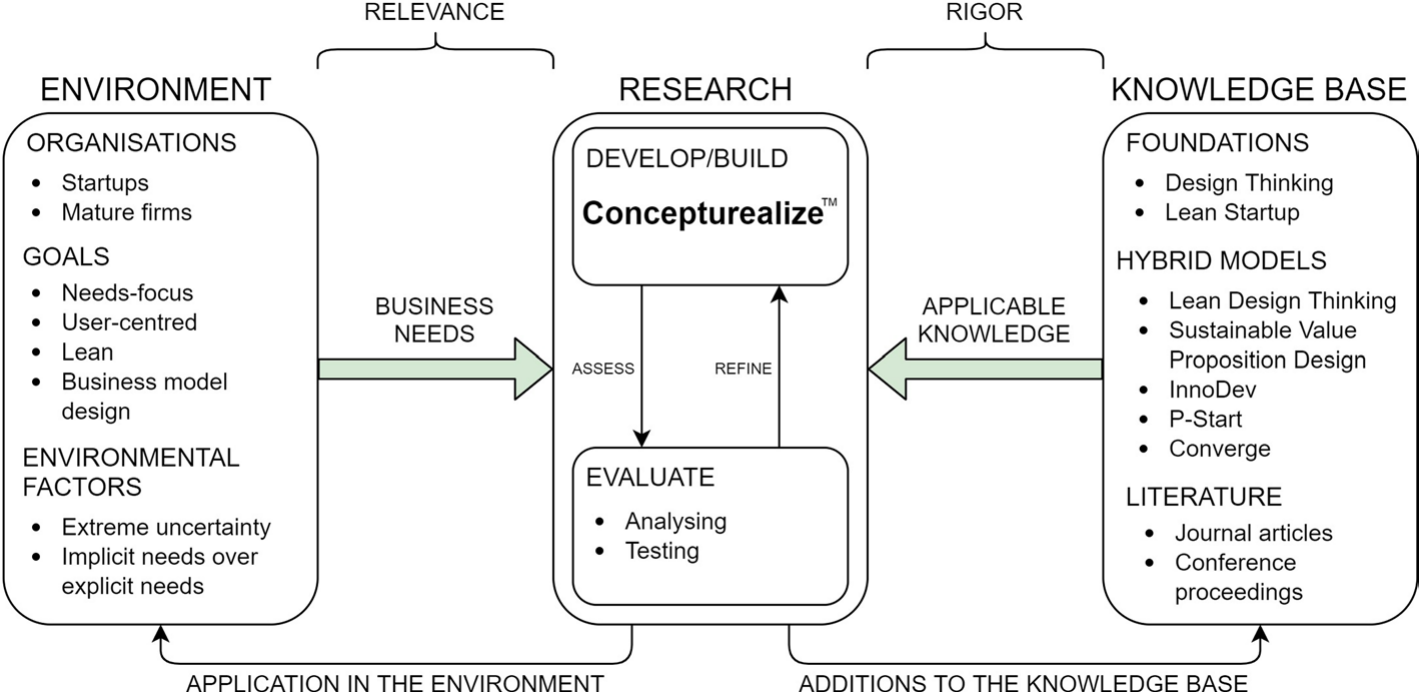
Application of design-science to the development of Concepturealize™ (adapted from Hevner & Park, 2004, p. 80). To satisfactorily answer the research question, first it was deconstructed to the following elements: **a** how can DT principles be combined into LS; **b** to generate real-needs-focussed; **c** user-centred; **d** lean; **e** business models?

The application of the Design-Science approach to the development of Concepturealize™ is illustrated in Fig. 4.

The five elements of the research question were then used to derive the purpose of Concepturealize™. Applicable knowledge, taken from DT and LS literature, previous attempts to combine models, and the wider literature review was used to specify the functions that Concepturealize™ must perform in order to achieve its purpose. Business needs, including consideration of organisational type, goals and environmental factors were used to inform the selection of the core tools to be used, within Concepturealize™, to perform the functions.

In the spirit of DT, a 2-year period of participative immersion in an entrepreneurial educational setting was conducted to help understand how entrepreneurship master’s degree students approach entrepreneurial problems. It was observed that from nine business planning projects (four projects in year 1, five projects in year 2), all groups, without exception, elected to use the Business Model Canvas (Osterwalder et al., 2005) to develop their respective business models, despite being enabled to select alternative approaches. As such, in order to maintain familiarity with LS and to aid the entrepreneur in achieving completeness of the business model being developed, the Concepturealize™ methodology was designed in such a way that it may be superimposed onto the Business Model Canvas (Osterwalder et al., 2005), as well as making use of tools frequently used in LS, such as the Value Proposition Canvas (Osterwalder et al., 2014), Five Whys and Innovation Accounting (Ries, 2011).

Following the initial specification of the functionality and the application of core tools, Concepturealize™ was constructed as an MVP (in the spirit of LS) and went through a series of BML feedback cycle iterations. The Concepturealize™ methodology was presented to a selection of nine target users, each familiar with LS and DT. The target users were selected according to their professional profiles with the intention of capturing a broad cross-section of users with differing specific interest in BMD (e.g. entrepreneurs, investors, business mentors and business professors). These target users included two CEOs of profitable, post-money startups; a co-founder of an early-stage pre-money startup; an innovation mentor; a head of entrepreneurship and startup support (Venture Capital); a business mentor at a national governmental economic development agency; and three university professors specialising in entrepreneurship and business model development. The target users were exposed to the Concepturealize™ methodology at various stages of its development, depending on their profiles’ expected purpose of interaction with the methodology (e.g. using the methodology for BMD, validation of existing business models, or disseminating or teaching the methodology).

Subject-matter experts in BMD, such as academics and business mentors, were brought into the development of the methodology early in the process, whilst the target users expected to have a more superficial level of exposure, such as entrepreneurs and investors, were exposed to the methodology for the first time towards the end of its development.

These target users were asked to provide commentary on the benefits they perceive and the difficulties that they foresee in relation to utilising the methodology as well as suggestions for improvement. The input from these target users was used for honing and refining the methodology, during the iterative process, to ensure applicability to the target environment, integrity of the toolset selected, and ease of use.

Target user feedback from the earlier iterations tended to have focus on the integrity and robustness of the toolset and had the effect of increasing practitioner load to ensure adequate coverage of all aspects of BMD. Later-stage feedback had greater focus on making the methodology simpler to follow, driving a reduction in practitioner load, whilst maintaining sufficient coverage of all aspects of BMD.

**Fig. 5:**
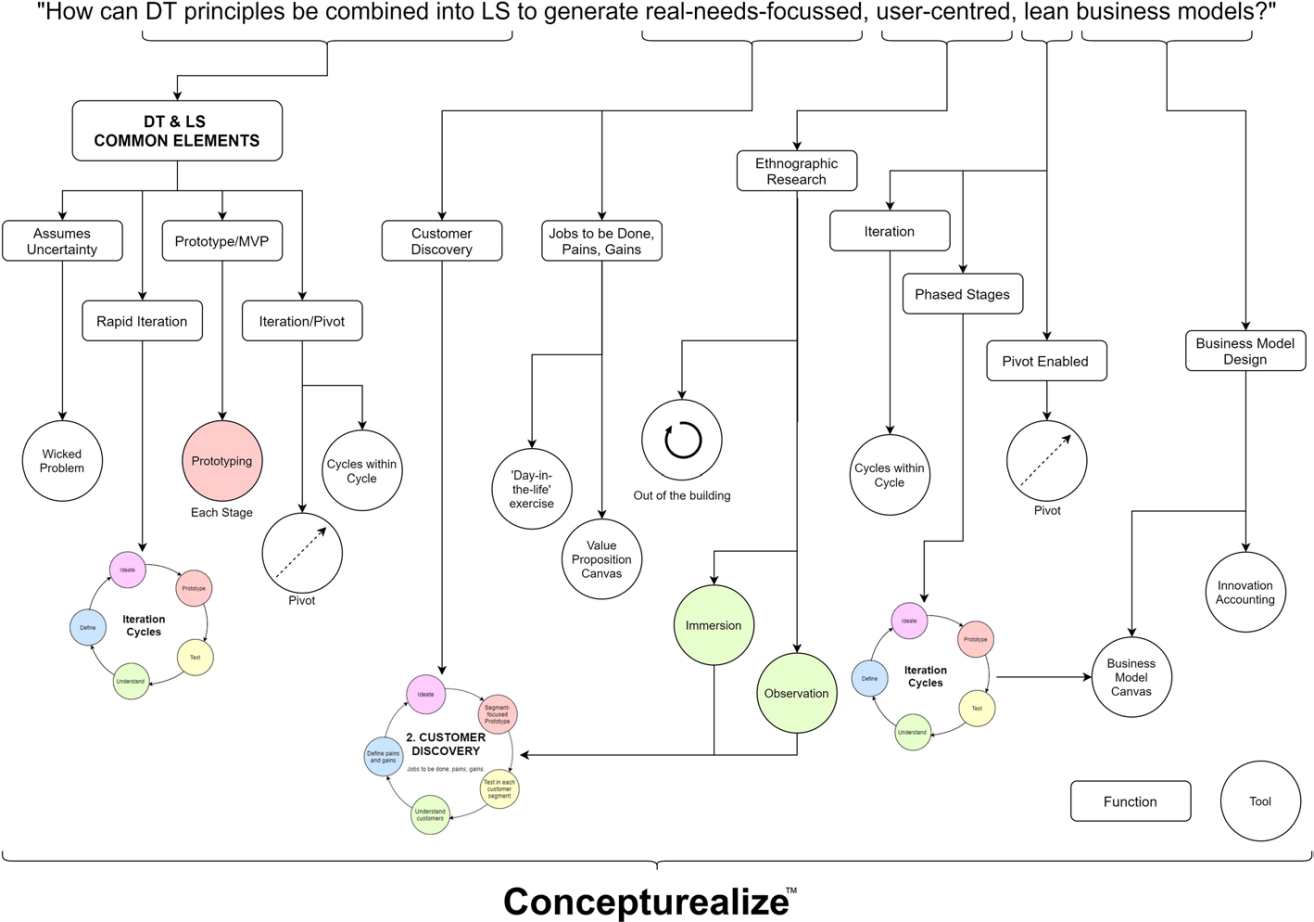
Concepturealize™ functions and core tools

Figure 5 represents the alignment of the elements of the research question, with the functionality of Concepturealize™, and the tools used to perform these functions.

## The Concepturealize™ methodology

The Concepturealize™ methodology assumes uncertainty, beginning with the search for ‘Wicked Problems’ by empathising with potential customers and observing and engaging with them to understand them on a psychological and emotional level. The methodology forms a cyclical process and further includes smaller sub-cycles, with the main process cycle and each sub-cycle being repeated, in an iterative manner, following a sub-step of ideation. Each step of the process includes the creation of a prototype artefact which is used for testing of hypotheses and to facilitate an understanding of the subject at hand. The process is strongly user-focussed with most steps designed to encourage the entrepreneur to ‘leave the building’ and interact with users/customers. Graphically, steps that require primarily ‘out of the building’ work are represented as a clockwise cycle, whereas steps that may be conducted ‘inside the building’ are shown as anti-clockwise cycles.

The methodology follows the LS framework, adding a DT cycle into each step. The whole process model can be superimposed onto the Business Model Canvas (Osterwalder & Pigneur, 2010). As the methodology is followed, each block of the BM canvas is explored, and outputs are generated using LS build–measure–learn- or DT-based sub-cycles. The process is a 10-step process (including step 0), as follows: (0) start; (1) observation; (2) customer discovery; (3) value propositions; (4) relationships and channels; (5) revenue streams; (6) key activities; (7) partners and resources; (8) cost structure; and (9) implementation. For simplicity and consistency, steps 1 to 8 each consist of five sub-steps arranged as a full cycle: ideating, prototyping, testing, understanding, defining. Step 0 (Start) is linear and is formed of three sub-steps: empathising, defining, ideating. Step 9 (Implementation) is a single step that encompasses the development and deployment of the MVP, product, or new features. The process model has built-in ‘pivot’ paths prescribed at Steps 2, 3, 6 and 8.

**Fig. 6:**
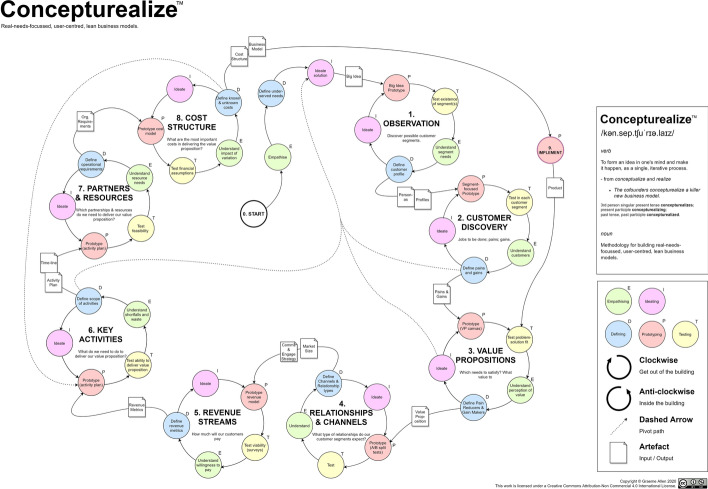
Concepturealize™ methodology

**Fig. 7:**
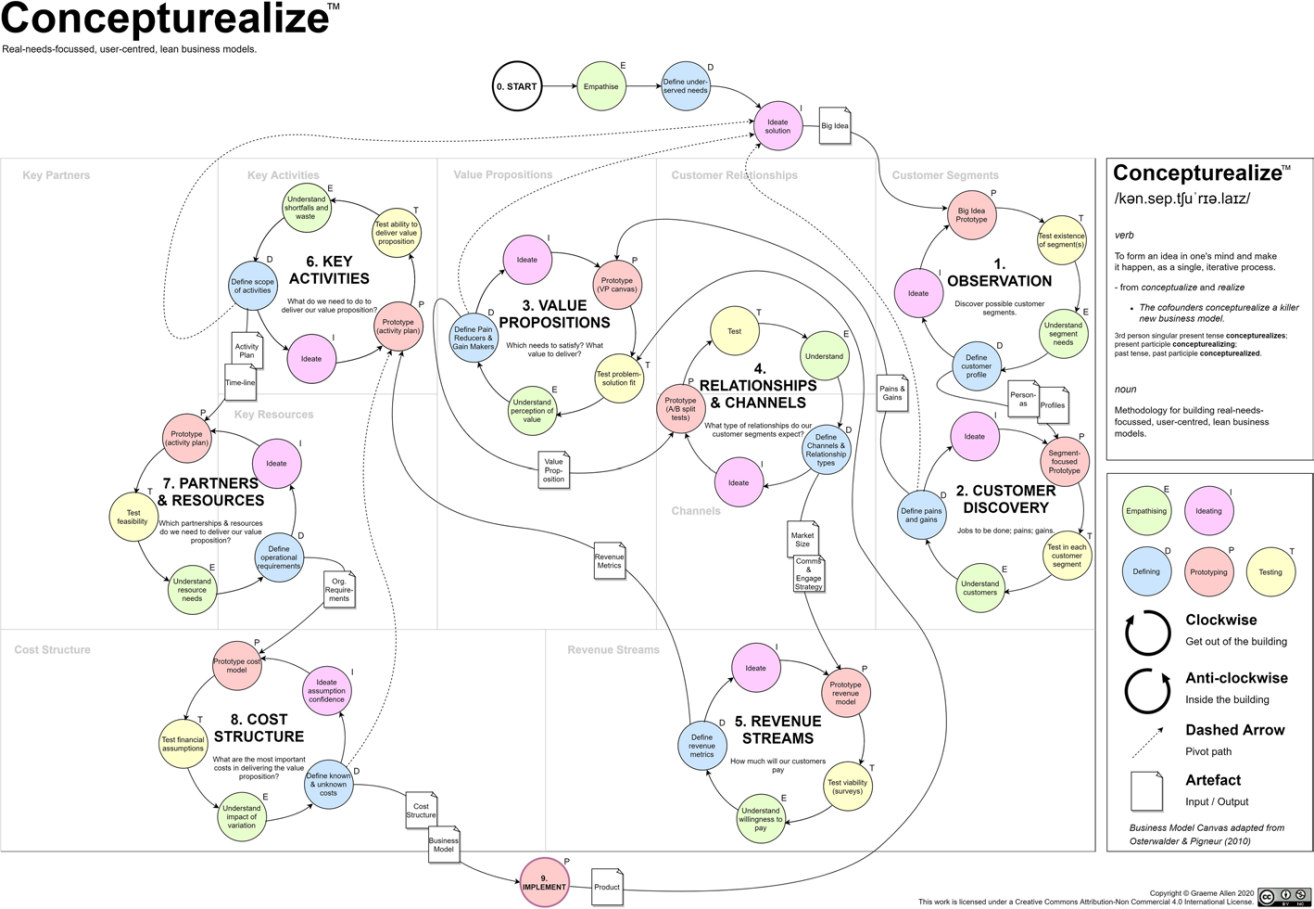
Concepturealize™ methodology, illustrated with business model canvas overlay based on Osterwalder and Pigneur (2010, p. 44)

An illustration of Concepturealize™ is shown at Fig. 6 and a graphical representation showing how the methodology overlays the business model canvas is shown at Fig. 7. A table showing all steps, including purpose, tools and outputs is shown as Table 5.

**Table 5:** Concepturealize™ steps

Step	Purpose	Tools	Outputs	Next Step
**0. Start**	**To identify "Wicked Problems"**		**Big Idea**	
a) Empathise	Observing and engaging with people to understand them on a psychological and emotional level	Immersion; Observation; Interviews	Broad understanding of existence of 'Wicked Problem(s)'	0b
b) Define under-served needs	To define the big user problem that your team needs to solve	5 Whys	Problem statement	0c
c) Ideate solution	To create your 'Big Idea' to address the wicked problem	Problem Statement (1b); Brainstorming; Mind mapping; Provocation	'Big Idea' to address problem	1a
**1. Observation**	**To discover existence of potential market**		**Customer profiles and personas**	
a) Big Idea prototype	To clearly explain 'Big Idea' to potential customers and elicit general feedback		Prototype (clear description of big idea)	1b
b) Test existence of segment	To ask, "Does the idea suitably address the problem?"	Prototype (1a); Surveys	Explicit feedback from potential customers	1c
c) Understand segment needs	To understand the needs of the customers/users in relation to the problem statement (for each segment, if more than one)	Prototype (1a); Immersion; Observation; Interviews	Implicit feedback from potential customers; Needs statement	1d
d) Define customer profile	1. To segment customers/users into groups defined by their needs 2. To create a customer profile for each segment type	Needs statement (1c)	Customer profile for each segment; Personas for each segment	If customer profile is fully defined: 2a; else: 1e
e) Ideate	To refine Big Idea in-line with customer/user feedback	Prototype (1a); Needs statement (1c); Customer profiles (1d); Personas (1d); Brainstorming; Mind mapping; Provocation	Improved 'Big idea' to address problem	1a
**2. Customer Discovery**	**To classify and prioritise market segments**		**Understanding of Customer Pains and Gains**	
a) Rough prototype	To demonstrate 'Big Idea' to target customer segment and elicit specific feedback	Needs statement (1c); Customer profiles (1d); Paper-prototypes; "Wizard of Oz" prototypes	Rough prototype that allows user to interact with your 'Big Idea'	2b
b) Test in each customer segment	To discover implicit needs (pains and gains) of each customer segment	Prototype (2a); Immersion; Observation; Interviews	List of pains and gains for each customer segment	2c
c) Understand customers	To understand the significance of the pains and gains of the customer segment	Prototype (2a); Immersion; Observation; Interviews	Understanding of the importance of the pains and gains that you are trying to address	2d
d) Define pains and gains	To define and prioritise the real (implicit) needs (pains and gains) of each customer segment		Prioritised list of pains and gains for each customer segment in relation to the problem being addressed	If pains and gains fully defined and significant: 3a; else if pains and gains fully defined and not significant: 0c; else: 2e
e) Ideate	To improve prototype or method to enable better elicitation of user-needs in relation to the problem statement	Needs statement (1c); Customer profiles (1d); Brainstorming; Mind mapping; Provocation	Improved prototype or method that allows user to interact with your 'Big Idea'	2a
**3. Value propositions**	**To define how you will create value for your customers**		**Value proposition(s)**	
a) Prototype	To describe your value proposition	VP canvas; Prioritised list of pains and gains (2d)	Filled Value Proposition Canvas for each customer segment; Value proposition statement	3b
b) Test problem–solution fit	To verify alignment between pains and pain relievers and gains and gain creators	VP canvas; Prioritised list of pains and gains (2d)	Focus on most important pain relievers and gain creators for creation of value for each customer segment	3c
c) Understand perception of value	To understand how your potential customers perceive value in your big idea	VP canvas; Day in the life exercise; Surveys; Immersion; Observation; Interviews	Qualification of the value, to each customer segment, of your big idea; Understanding of the importance of each of the identified pains and gains; Understanding of the ability of your value proposition to reduce pains and create gains	3d
d) Define pain reducers and gain creators	To refine value proposition to focus on the most important pain reducers and gain creators	VP canvas	VP canvas with value proposition(s) fully aligned with customer needs	If pain reducers and gain creators fully defined and align with pains and gains: 4a; else if pain reducers and gain creators fully defined and not aligned with pains and gains: 0c; else: 3e
e) Ideate	To improve your value proposition	VP canvas; Prioritised list of pains and gains (2d); Brainstorming; Mind mapping; Provocation	Updated Value Proposition Canvas	3a
**4. Relationships and Channels**	**To determine how to reach your customers and the kind of relationship(s) they expect**		**Knowledge of market size and reach; Communication and engagement strategy**	
a) Prototype	To test communication and customer engagement methods	Landing pages; Social media; Industry data	Provisional communication and engagement strategy; Prototype elements (could be a web landing page, social media page, podcast, tradeshow stand, etc.)	4b
b) Test	To verify efficacy and potential reach of channels	Web analytics; A/B split testing; Industry data; Interviews with channel partners; Surveys	Quantification of potential reach of channels for communication of your value proposition	4c
c) Understand	To understand how your customers can be reached and the type of relationships they expect	Prototype (4a); Immersion; Observation; Interviews with customers	Qualification of customer relationship types for delivery of your value proposition	4d
d) Define relationships and channel types	To define your potential market reach and strategy to achieve it	Growth funnel (Acquisition, Activation, Retention, Referral and Revenue (AARRR))	Size of market and reach potential (TAM, SAM, SOM); Communication and engagement strategy	If relationships and channels fully defined and fit value proposition: 5a; else: 4e
e) Ideate	To improve size of market and reach potential (TAM, SAM, SOM); To refine communication and engagement strategy	Brainstorming; Mind mapping; Provocation; Landing pages; Social media; Industry data	Improved prototype inputs or method for testing of communication and engagement strategy	4a
**5. Revenue Streams**	**To understand your required and expected revenue streams and the impact of their variation**		**Revenue metrics and risk profile**	
a) Prototype	To model baseline revenue forecasts	Innovation Accounting; Sales forecast; Income statement; Cash flow forecast; Balance sheet; P&L forecast	Revenue model	5b
b) Test viability	To understand impact of variation	Innovation accounting; Traditional financial forecasting and modelling methods	Understanding of the impact of variation of revenue	5c
c) Understand willingness to pay	To understand likelihood of variation	Industry data; Interviews with channel partners; Competitor research; Interviews with customers	Understanding of the likelihood of variation of revenue; Validation of forecasted revenue	5d
d) Define revenue metrics	To define revenue metrics, willingness of the customer to pay, and associated risk	Innovation accounting; Forecasting	Defined, validated revenue metrics; Revenue risk profile	If revenue metrics and customer willingness to pay fully defined: 6a; else: 5e
e) Ideate	To improve modelled revenue forecasts	Revenue model; Brainstorming; Mind mapping; Provocation	Improved revenue model inputs	5a
**6. Key Activities**	**To define the activities required to deliver the value proposition**		**Activity plan and timeline**	
a) Prototype	To record activities required to deliver value proposition		Activity plan	6b
b) Test ability to deliver value proposition	To identify whether the activities deliver each element of the value proposition, including all pain relievers and gain creators, as well as facilitating channels and customer relationships and business activities such as accounting, HR and legal	Activity plan (6a); value proposition canvas (3d); Communication and engagement strategy (4d); Revenue metrics (5d)	Full list of required activities	6c
c) Understand shortfalls and waste	To understand which necessary activities are unprovided or unnecessary activities are included	Activity plan (6a); value proposition canvas (3d); Communication and engagement strategy (4d); Revenue metrics (5d)		6d
d) Define scope of activities	To define and prioritise all necessary activities and their relationships with business operations and deliverables; To define sequence and timeline of activities	Activity plan (6a); value proposition canvas (3d); Communication and engagement strategy (4d); Revenue metrics (5d)	Prioritised activity list; Activity relationship plan; Project plan/timeline	If scope of activities fully defined and delivers value proposition and feasible: 7a; else if scope of activities fully defined and not feasible: 0c; else: 6e
e) Ideate	To identify how to ensure delivery of value proposition whilst minimising activities and eliminating waste	Activity relationship plan; Project plan/timeline ; Brainstorming; Mind mapping; Provocation	Improved activity plan inputs	6a
**7. Partners and Resources**	**To understand which partnerships & resources are needed to deliver the value proposition**		**Operational resources and requirements plan**	
a) Prototype	To identify which resources and partnerships are needed to complete all activities	Activity plan	Activity relationship plan; Project plan/timeline with resources allocated;	7b
b) Test feasibility	To uncover which activities can be fulfilled with exiting or planned resources, and which need to be outsourced			7c
c) Understand resource needs	To understand the advantages and disadvantages of adding resources or outsourcing for each activity	Project plan; Risk analysis; SWOT analysis	Requirements analysis	7d
d) Define operational requirements	To define operational strategy		Operational resources and requirements	If operational requirements for partners and resources fully defined: 8a; else: 7e
e) Ideate	To improve operational strategy by playing on strengths and reducing risk	Brainstorming; Mind mapping; Provocation	Improved activity relationship and resource plan inputs	7a
**8. Cost Structure**	**To understand the costs in delivering the value proposition**		**Business model**	
a) Prototype	To model baseline cost forecasts	Innovation Accounting; Sales forecast; Cash flow forecast; Balance sheet; P&L forecast	Cost model	8b
b) Test financial assumptions	To test reliability of assumptions	Industry data; Enquiries with suppliers; Competitor research	Understanding of the likelihood of variation of costs	8c
c) Understand impact of variation	To understand impact of variation	Innovation accounting; Traditional financial forecasting and modelling methods	Understanding of the impact of variation of costs	8d
d) Define known and unknown costs	To define financial cost metrics and associated risk to viability of delivery of value proposition	Innovation accounting; Forecasting	Defined, validated cost metrics; Cost risk profile; Business model	If known and unknown costs fully defined and viable: 9a; else if known and unknown costs fully defined and not viable: 6a; else: 8a
e) Ideate	To improve modelled financial cost forecasts and reduce risk	Assumption confidence	Improved inputs to cost model	8a
**9. Implementation**	**To deploy the MVP or added feature**		**Product**	
a) Development and Deployment	To develop and deploy MVP or added feature in-line with business model (8d)	Resources; Partnerships; Business model (8d)	Product	3b

## Results and discussion

The Concepturealize™ methodology was evaluated, according to the design-science approach (Hevner & Park, 2004), by use of descriptive evaluation through informed argument (by building upon existing artefacts with demonstrated utility), by demonstrating utility through a detailed scenario, and by analytical evaluation through examination of artefact structure and elements for static qualities (comprehensiveness and applicability to the problem, integrity of the toolset, familiarity of individual tools to target users, and ease of use).

### Scenario

The imagined scenario presented demonstrates the utility of Concepturealize™ by following a fictitious practitioner through the complete methodology. The practitioner should be considered as a new entrepreneur at the very beginning of conceiving a new startup venture, not having identified a problem to address. The location and industry of the startup, together with the background and core-skills of the practitioner, are intentionally undefined to aid demonstration of the generality of the methodology, although the practitioner having a working knowledge of DT and LS practices is assumed.

**Step 0: Start. **The process begins at Step 0 with the purpose of identifying ‘Wicked Problems’. The practitioner starts by empathising with people by observing and engaging with them to try to understand them on a psychological and emotional level. The practitioner uses immersion and observation and realises that people appear to dislike getting wet when it rains. They then use interviews to discover the reasons that people dislike getting wet include an aversion to feeling cold and not wanting to present a dishevelled appearance. Next, the practitioner defines the under-served needs that they have uncovered through the empathic understanding, they define the big user problem that needs to be solved, using tools such as 5-whys. In this scenario, the practitioner discovers that people would prefer to stay indoors when it is raining but often need to go outdoors, despite the rain, to travel to work or run errands. Finally, they ideate to create a ‘Big Idea’ to address the under-served needs, using brainstorming and mind-mapping techniques.

**Step 1: Observation.** Following step 0, the practitioner enters Step 1. Here, the purpose is to discover the existence of a potential market for the big idea. Step 1 is a cyclical sub-process that starts with a ‘big idea prototype’—simply a clear and concise description of the big idea. In this scenario, the big idea is ‘a lightweight portable roof that the user can wear upon their head to keep themselves dry’. The practitioner tests the big idea by describing it to potential customers and eliciting feedback through surveys or other forms of quantitative research. The practitioner discovers that many people do not feel that they would use such a contraption.

Next, the practitioner develops deeper understanding by building on the quantitative data to understand the implicit needs of the potential customers, by methods such as interviews. In this scenario, our practitioner learns that many people would feel self-conscious about their appearance when wearing such a device upon their heads, while others are only concerned about the inconvenience when negotiating tight spaces, such as alleyways. The practitioner uses the insights they gain to generate a needs statement and customer profiles, and to segment users into groups, based on those needs (in this scenario, the people that give more importance to appearance, and those that give more importance to utility and convenience). The practitioner must then use the newly acquired better understanding of the customers and their needs to improve the big idea before retesting and developing even greater understanding, iterating until the big idea can no longer be significantly improved. At this point, the practitioner moves to step 2.

**Step 2: Customer discovery.** Using the needs statement and profiles generated in Step 1, the practitioner generates a simple prototype to allow target customer representatives to interact with the big idea, this is a simple paper-prototype or a ‘Wizard of Oz’ prototype,^5^ perhaps a mock-up of an application or an analogous representation of a product idea. In this case, the prototype is a plastic dustbin lid affixed to an open-faced motorcycle helmet. The prototype is tested within each segment and used to gain insight of the implicit ‘pains and gains’ in relation to the big idea. Our practitioner discovers that the inconvenience of wearing such a thing on one’s head outweighs the pains the come from being wet from the rain.

The following sub-step is to understand the customers—to gain a deeper understanding of the importance of the pains and gains (i.e. not just to know the pains or gains exist, but to understand why they exist and how important they are to the customer). The insights gathered from this sub-step are then used to define and prioritise the pains and gains for each segment for use in improving the prototype or testing method to be used in the next iteration of the cycle. Our practitioner begins to understand that an important relevant pain for older people is the fear of becoming ill from spending time in wet clothes—however, this same group of people are frequently concerned with the risk of injury caused by a gust of wind catching the headwear whilst it is in use—if this risk could be eliminated, they would use the product.

Our practitioner iterates on the prototype, eventually affixing the dustbin lid to the end of a rod so that it may be held above the head with one hand, rather than it being attached to the user’s head. At this point, user feedback indicates that the device suitably relieves the pain associated with being wet from the rain. Further feedback indicates that some users that live in drier climates perceive an additional gain from using the product to shade themselves from the sun. Our practitioner further discovers that, in general, users require the device to be lightweight and to be foldable for easy handling and storage when not in use. One user suggests that the device be fitted with lighting to assist when walking at night.

Once improvements can no longer be realised, and assuming the pains and gains have been fully defined as significant enough to warrant further exploration, the practitioner moves to step 3. If the pains and gains, in relation to the big idea, are defined as not being significant enough to warrant further exploration, the practitioner should ‘pivot’ by returning to Step 0 to come up with a new big idea. In this scenario, the pains and gains are defined as significant, so the practitioner moves to Step 3.

**Step 3: Value proposition.** This step begins with the ‘Value Proposition Canvas’ (Osterwalder et al., 2014) as the prototype. Our practitioner uses the canvas to test the problem–solution fit—to align the pain relievers and gain creators from the big idea with the pains and gains observed in Steps 1 and 2.

Our practitioner then begins to understand the wider perception of value by using tools such as day-in-the-life exercises, surveys, immersion, observation, and interviews to understand how a wider sample of potential customers perceive value in the big idea; and fully defines the pain relievers and gain creators and how they align with customer needs. Finally, the practitioner iterates the value proposition by further ideation and repetition of the previous sub-steps. Once the pain relievers and gain creators provided by the big idea have been fully defined, the practitioner moves on to Step 4, or returns to Step 0 should it not be possible to align the value proposition with the needs of the customer. In this scenario, our practitioner defines that the gain provided by built-in lighting is not significant for most users, so the feature is dropped from the value proposition. The lightweight and foldable properties of the product are significant so are retained. The pain reducers and gain creators provided by the product are now aligned with the most significant pains and gains of the customer, so our practitioner moves to Step 4.

**Step 4: Relationships and channels.** Step 4 covers the channels through which the customers may be reached and the types of relationships the business will have with them. This step follows a similar five sub-step cycle, making use of tools such as web analytics, A/B split testing,^6^ industry data, interviews with channel partners, surveys, and interviews with customers to define the size of the potential market and reach potential (Total Addressable Market (TAM), Serviceable Addressable Market (SAM), Serviceable Obtainable Market (SOM)) as well as a communication and engagement strategy. The practitioner continues to iterate this step until no further improvement is realised before moving to Step 5. Here, our practitioner defines that the market size is attractive, and that homeware, clothing and sporting goods retailers would stock such a product. Further, our practitioner iteratively develops their initial marketing strategy, opting to start with online sales and to later develop a market through high street retailers.

Note that, in contrast to the Business Model Canvas (Osterwalder & Pigneur, 2010), where Customer Relationships and Channels are approached as separate blocks, Concepturealize™ encourages the practitioner to consider them together. A particular channel may improve, or indeed prohibit, a particular relationship type (and a particular relationship type may improve or prohibit a particular channel)—considering both elements in unison enhances the fit between them.

**Step 5: Revenue streams.** Step 5 exists to understand the required and expected revenue streams and the impact of their variation on the deliverability of the value proposition. This step uses tools such as innovation accounting (Ries, 2011), sales forecasting, income statement projections, and cash flow forecasting, together with interviews with channel partners, competitor research and interviews with customers, to understand and define revenue metrics and risks. Again, a five sub-step cyclical process is used here. Once improvement through iteration has been exhausted, the practitioner should move to Step 6. Our practitioner now understands the customers’ willingness to pay, the maximum acceptable retail price and the expected sales margin from the retailers.

**Step 6: Key activities.** At Step 6, the practitioner should come to understand, and be able to define, the activities required to deliver the value proposition. The sub-steps are to prototype the activity plan; test the ability of the identified activities to deliver the value proposition; understand where there are shortfalls or wasted activities; define a full scope of activities, with a project plan or timeline; and then exhaust the iteration cycle before moving on. If it is found that the activities required to deliver the value proposition are not feasible, the practitioner should pivot by returning to Step 0 to come up with a new big idea for which a feasible value proposition could be devised. Otherwise, they should move to Step 7. In this scenario, our practitioner iterates though the activity plan until they have fully defined the main activities of product design and engineering, production, warehousing, marketing, and sales, as well as all of the foreseeable supporting activities including staffing, accounting, contract management, etc.

**Step 7: Partners and resources.** Now that the practitioner understands the activities that mut be conducted in order to deliver the value proposition, they may begin to understand what resources and partnerships they will need to complete the activities. This is Step 7. The practitioner conducts risk analyses, and strengths, weaknesses, opportunities, and threats (SWOT) analyses, in the context of the project plan and activity plan from Step 6, to understand how best to organise the resources and which partnerships to best pursue in order to be able to deliver the value proposition. Here our practitioner again iterates through the activity plan, allocating resources or partners, as appropriate. They decide to outsource all activities, except for managing the startup, to external companies or consultants.

Note that, in contrast to the Business Model Canvas (Osterwalder & Pigneur, 2010), where Key Partners and Key Resources are approached as separate blocks, Concepturealize™ encourages the practitioner to combine them. Each activity defined in Step 6 must be performed either by a partner or by a resource, else it is not performed at all. Giving consideration to partners and resources, in unity, decreases the chances of an activity not being covered by either.

**Step 8: Cost structure.** With an understanding of the revenue model and the activities that must be conducted, together with an understanding of who will conduct them (i.e. which activities are handled by internal resources, and which are handled by partners), the practitioner may move to Step 8. By use of innovation accounting techniques and traditional financial forecasting and modelling methods, the practitioner should build a full picture of the expected cost structure. Industry data, enquiries with suppliers and competitor research should be used to inform the model, which is then used to identify and understand the reliability of the assumptions made and the impact of any variation. Once iterative improvement of the cost structure ceases to yield results, this step outputs cost metrics and completes the business model and the practitioner may move to Step 9 (Implementation). If, however, it is found that the cost of the required activities makes the cost model non-viable, the practitioner should pivot by returning to Step 6, where they will redefine the activities required to deliver the value proposition, and subsequently, the partnerships and resources required to conduct the activities. If the costs of delivering the value proposition are still not viable, the practitioner should pivot by returning to Step 0 (via Step 6) and repeat all steps to discover a viable way to create value.

In this scenario, our practitioner learns that it is not feasible to deliver the value proposition within the available budget. As such they return to Step 7 and reiterate through the activity plan, removing warehousing, instead opting for just-in-time production. The practitioner carries the revised activity plan through Step 8, redefining product design as an activity to be conducted by an internal resource, opting to recruit an experienced product manager to the team. Finally, our practitioner reiterates through Step 8. It is now feasible to deliver the value proposition within the available budget, so they move to Step 9.

**Step 9: Implement.** Now that our practitioner has followed steps 0 to 8 and uncovered a viable way to create value, developed a deep understanding of the value proposition, the target customers and how to reach and serve them, together with the expected revenue and costs, they develop and deploy the product that will deliver the value proposition, within the parameters of the business model.

After deployment, the practitioner returns to Step 3 and retests the problem–solution fit and understands how the customers perception of value has changed since the implementation of the product—following through all subsequent steps, looking to continually add value at each cycle.

### Static analysis

The methodology was presented to target users (without personal connections to the author) within three organisation types, who were each asked to provide critical feedback. The target users’ profiles were the CEO and founder of a profitable, post-money startup (user 1); a head of entrepreneurship and startup support, and business mentor, at a national governmental economic development agency (user 2); and an innovation mentor and professor at a state university (user 3). All three agreed that Concepturealize™ offers value to entrepreneurs, with user 1 commenting that “[Concepturealize™] touches all the key aspects to reflect about when implementing business models and assure that they bring the right revenue stream”, but that “finding a good fit problem–solution–value proposition-business model sometimes does not suffice, as it is necessary to educate the market and promote, which consume time and money and that is not considered in [Concepturealize™]”. To address this, further detail was added to the description, particularly around ‘Step 4: Relationships and Channels’ and ‘Step 8: Cost Structure’ to enhance clarity around how marketing and promotion form part of the BMD process.

User 2 reported that “[Concepturealize™] presents a logical framework that can give a constructive and progressive format to something which is abstract and surrounded by unknowns” and that “it is well broken-down and allows for identifiable steps for each stage; it allocates each phase its respective degree of importance and also allows for the new entrepreneur to [pivot], if necessary”.

User 3 reported that they found Concepturealize™ to be a new methodology which offers a “synergistic process, clear criteria, and clear relationships”.

Suggestions for improvement included adding elements based on the type of business (for example business-to-business, business-to-consumer, business-to-business-to-consumer, etc.); and further development of the model into a ‘virtual assistant’ or ‘chat bot’ that could provide hints and warnings to the practitioner.

Other feedback included a request for the inclusion of a version of the Concepturealize™ illustrated flow without the BM canvas underlay, as the respondent felt that the methodology overlayed on the BM canvas may ‘scare’ new users. This feedback was actioned by providing the version of the illustration shown as Fig. 6.

## Conclusions

The question that this work aimed to answer is, “how can Design Thinking principles be combined into Lean Startup to generate real-needs-focussed, user-centred, lean business models?”.

Concepturealize™ answers the research question by presenting a novel methodology that cross-applies DT and LS and that enables the practitioner to generate real-needs-focussed, user-centred, lean business models—achieving research objective 1—and that improves on the independent use of both DT and LS, in the context of BMD, whilst retaining the lean nature of LS and the user-centredness of DT—achieving research objective 2.

The research began with an in-depth literature review to identify and classify previous attempts to cross-apply DT or LS with each other or with other models. The studies were classified according to the level of testing rigour, e.g. whether used in real-world case studies, etc.; the level of success of the model; and where available, evidence of adoption of the model, post-study. The literature revealed that there have been several attempts to develop new process models that integrate DT and LS, either with each other, or with other methodologies or models. However, there appears to be a need for further exploration of cross-application of DT and LS (and other related methodologies) in the areas of business model development and innovation.

Following the literature review, the most developed and tested models were used as a foundation to produce a new viable methodology as a working artefact. The lessons learned from the previous attempts, as well as the literature pertaining to DT and LS, and other relevant models, were used to guide the formation of the Concepturealize™ methodology.

Concepturealize™ begins with the search for ‘Wicked Problems’ by empathising with potential customers and observing and engaging with them to understand them on a psychological and emotional level. The process model is a cyclical model and further includes smaller sub-cycles, with the main process cycle and each sub-cycle being repeated, in an iterative manner, following a sub-step of ideation. Each step of the process includes the creation of a prototype artefact which is used for testing of hypotheses and to facilitate an understanding of the subject at hand. The process is strongly user-focussed with most steps designed to encourage the practitioner to ‘leave the building’ and interact with users/customers.

By following the process correctly, the entrepreneur will be guided to uncover a viable way to create value, develop a deep understanding of the value proposition, the target customers and how to reach and serve them, together with the expected revenue and costs, all needed to properly formulate the business model. Finally, the entrepreneur should use the Concepturealize™ methodology to retest the problem–solution fit and understand how the customers perception of value has altered, each time a new product or new features are launched, looking to continually add value at each cycle.

### Contributions

Whilst prior research has explored how organisations may make use of both DT and LS, it has failed to demonstrate how they may be used in parallel, throughout the entire business model development process, instead it demonstrates examples of insight into where to transition from one model to the other. This work progresses the state of the art by demonstrating how the true, in-parallel, cross-application of DT and LS, in the context of business model development, is possible.

### Implications for practice

Concepturealize™ has positive implications in helping entrepreneurs to develop innovative and sustainable business models in a lean, real-needs-focussed, user-centred manner. It improves on the use of LS, independently, by increasing the likelihood of proper consideration being given to the superiority of other ideas, whilst retaining the ability to achieve a short time to market. Further, Concepturealize™ improves on the independent use of DT by providing tools to increase efficiency in execution and commercialisation. Concepturealize™ improves on the hybrid methodologies and models identified in the literature review by truly integrating DT into LS and by its dynamic nature (provided by built-in pivot loops), emphasis on user-centredness, and by increased flexibility thorough comprehensive use of iteration (applying iterative sub-cycles to each element, within an iterative master-cycle), to the entire business model.

### Limitations and future research

At the time of this work being conducted, the world’s community was working to stem the spread of a global virus pandemic (COVID-19), with non-essential workers in many countries in lockdown. Therefore, it was not feasible to test the methodology within a live scenario—particularly due to the strong focus of DT on group-collaboration and ethnographic activities, such as immersion and observational studies, and the emphasis of LS on ‘getting out of the building’.

The Concepturealize™ methodology was designed in such a way, and presented within this report, both graphically and with all steps tabulated, showing each step together with purpose, tools, and outputs. This allows for later testing of the methodology within business organisations, startups, or entrepreneurship training courses once the global community re-establishes ‘business as usual’. It is recommended that the Concepturealize™ methodology be deployed for testing and validation within such settings.

The scope of this work was limited to DT and LS, as such, it does not explore the cross-application with other methodologies or frameworks, in depth. Additional benefit could be gained by further exploration and identification of tools most suited to the various steps and sub-steps of the Concepturealize™ methodology as well as further research into other methodologies or frameworks suited for cross-application.

There is an opportunity for future work to explore how the methodology may be adapted (if at all) to the type of business (for example business-to-business, business-to-consumer, business-to-business-to-consumer, etc.).

## Data Availability

The datasets used and/or analysed during the current study are available from the corresponding author on reasonable request.

## Abbreviations

AARRR: Acquisition, activation, retention, referral and revenue
BM: Business model
BMD: Business model design
BML: Build–measure–learn
DT: Design Thinking
LS: Lean Startup
MVP: Minimum viable product
NPD: New product development

## Footnotes

1 IDEO is a global design and consulting firm that uses the design thinking approach, extensively.

2 Methodology for building startups and new corporate ventures, developed by Steve Blank.

3 Methodology for minimising waste, developed by Taiichi Ohno and Shigeo Shingo at Toyota.

4 Model for New Product Development created by Robert G. Cooper.

5 A research experiment in which subjects interact with a system that they believe to be autonomous, but which is actually being operated an unseen human being.

6 A user experience research methodology consisting of a randomised experiment with two variants.
